# *Aspergillus niger* citrate exporter revealed by comparison of two alternative citrate producing conditions

**DOI:** 10.1093/femsle/fnz071

**Published:** 2019-04-10

**Authors:** Dorett I Odoni, Marta Vazquez-Vilar, Merlijn P van Gaal, Tom Schonewille, Vitor A P Martins dos Santos, Juan Antonio Tamayo-Ramos, Maria Suarez-Diez, Peter J Schaap

**Affiliations:** 1 Laboratory of Systems and Synthetic Biology, Wageningen University & Research, Stippeneng 4, 6708 WE Wageningen, The Netherlands; 2 International Research Center in Critical Raw Materials-ICCRAM, Advanced Materials, Nuclear Technology and Applied Bio/Nanotechnology, University of Burgos, Plaza Misael Bañuelos s/n, 09001 Burgos, Spain

**Keywords:** *Aspergillus niger*, citrate, transport, transcriptomics, homology, MDR

## Abstract

Currently, there is no consensus regarding the mechanism underlying *Aspergillus niger* citrate biosynthesis and secretion. We hypothesise that depending on the experimental setup, extracellular citrate accumulation can have fundamentally different underlying transcriptomic landscapes. We show that varying the amount and type of supplement of an arginine auxotrophic *A. niger* strain results in transcriptional down-regulation of citrate metabolising enzymes in the condition in which more citrate is accumulated extracellularly. This contrasts with the transcriptional adaptations when increased citrate production is triggered by iron limitation. By combining gene expression data obtained from these two very distinct experimental setups with hidden Markov models and transporter homology approaches, we were able to compile a shortlist of the most likely citrate transporter candidates. Two candidates (An17g01710 and An09g06720m.01) were heterologously expressed in the yeast *Saccharomyces cerevisiae*, and one of the resultant mutants showed the ability to secrete citrate. Our findings provide steps in untangling the complex interplay of different mechanisms underlying *A. niger* citrate accumulation, and we demonstrate how a comparative transcriptomics approach complemented with further bioinformatics analyses can be used to pinpoint a fungal citrate exporter.

## Teaser

A comparative transcriptomics approach to disentangle*Aspergillus niger* citrate production is combined with other bioinformatics approaches to pinpoint a citrate exporter, which is validated by heterologous expression in *Saccharomyces cerevisiae*.

## INTRODUCTION

*Aspergillus niger* citrate production is a notorious example of a process that requires a unique combination of unusual nutrient and environmental conditions (Karaffa and Kubicek 2003). Carbon excess relative to iron, zinc, copper, manganese, phosphorus, magnesium, potassium and nitrogen reportedly all leads to an increase in *A. niger* citrate secretion in the form of either absolute yield or relative productivity (Chesters and Rolinson 1951; Karaffa and Kubicek 2003). The result of this is that although *A. niger* citrate production has been subject to study since Curries fundamental breakthroughs regarding *A. niger* citrate fermentation more than 100 years ago (Currie 1917), it is still not fully understood.

One problem might be that there are multiple factors at play, each independently or in combination influencing citrate secretion, and studies, therefore, contradict each other depending on which aspect or time point of citrate accumulation was investigated (Karaffa and Kubicek 2003). Citrate secretion under conditions of high glucose concentrations in the medium is generally accepted to be the result of overflow metabolism, i.e. excess flux through glycolysis and ultimately the TCA cycle, leading to the ‘undesired’ (from the perspective of the fungus) accumulation of citrate (Legiša and Mattey 2007). Nevertheless, the viewpoint that citrate is solely an overflow metabolite is changing, and *A. niger* citrate secretion might also be regarded as a response to environmental conditions such as competition (Andersen *et al*. 2011), or low iron bioavailability (Odoni *et al*. 2017). Further understanding of the various aspects that lead to increased citrate production in *A. niger* can provide tools for metabolic engineering approaches to further control and modulate citrate production.

An example tool for the control and modulation of citrate production can be found at transporter level (Karaffa and Kubicek 2003). It has been shown that overexpressing or introducing specific transporters for the product of interest can often overcome product limitation (van der Straat and de Graaff 2014). Dynamic models of metabolism have highlighted the citrate exporter as one of the proteins whose overexpression could lead to increased citrate production rates (Alvarez-Vasquez, González-Alcón and Torres 2000). The citrate transport system in *A. niger* has been described (Netik *et al*. 1997), and a comprehensive list of putative citrate transporter candidates in *A*. niger has been compiled using a transcriptomics approach (Yin *et al*. 2017), but thus far, gene expression analyses failed to pinpoint a definite *A. niger* citrate exporter.

Here, we explore how combining multiple gene expression datasets with other bioinformatic approaches can narrow down the list of putative citrate exporter candidates. For this, we worked with well-defined *A. niger argB* knock-out mutants. The *∆argB* mutation induces an arginine auxothropy that can be overcome by media supplementation with either arginine or citrulline (Lenouvel, van de Vondervoort and Visser 2002). We studied the impact of supplement type and amount on citrate production and performed transcriptome analysis to pinpoint the metabolic adaptations associated with the higher citrate producing condition. We compared the results of this study with previous data regarding changes in citrate production triggered by low iron concentrations in the medium (Odoni *et al*. 2017). Expression data were combined with hidden Markov models (HMMs) and homology approaches to compile a shortlist of the most promising citrate transporter candidates. We validated our approach by heterologously expressing two selected citrate exporter candidates in *Saccharomyces cerevisiae*, and show that one of these yeast transformant strains indeed accumulated a measurable amount of extracellular citrate.

## MATERIALS AND METHODS

### Strains and media

*Aspergillus niger* strains NW305 (*cspA, goxC17, ∆argB*) (Ruijter *et al*. 2003), and NW186 (*cspA1, goxC17, prtF28, ∆argB*) - a derivative of NW185 (Ruijter, Van De Vondervoort and Visser 1999) with restored uridine prototrophy (Odoni *et al*. 2017) - were used for identification of putative citrate exporter candidates. Strains were maintained on complete medium (CM) as described in Odoni *et al*. (2017). *Aspergillus niger* spores were harvested with Saline-Tween (0.9% NaCl and 0.001% Tween80).

*Escherichia coli* DH5α was used for standard gene cloning purposes. *Escherichiacoli* was cultured in Luria broth (LB) medium (5 g·L^−1^ yeast extract, 10 g·L^−1^ peptone, 10 g·L^−1^ NaCl) supplemented 100 mg·L^−1^ ampicillin when necessary.

*Saccharomyces* cerevisiae strain CENPK2–1D (MATα; his3D1; leu2–3_112; ura3–52; trp1–289; MAL2–8c; SUC2) was used for validating the citrate transporter candidates. Preparation of CENPK2–1D yeast electro-competent cells and transformation was performed as described in Suga and Hatakeyama (2003). Yeast-transformed cells were selected in synthetic media (SD) with 2% (w/v) dextrose, 0.67% (w/v) Yeast Nitrogen Base without amino acids (BD), 0.14% (w/v) Yeast Synthetic Drop-out Medium supplement without uracil, tryptophan, histidine and leucine (Sigma-Aldrich), 0.0076% (w/v) histidine, 0.0076% (w/v) tryptophan and 0.038% (w/v) leucine. For *S. cerevisiae* transformation, the cell suspension was mixed with plasmid or linear DNA, transferred to a pre-chilled cuvette (0.2 cm Gene Pulse, Bio-Rad, Uden, The Netherlands), pulsed at 2.5 kV, 25 μF, 200 Ω using Gene Pulser Xcell (Bio-Rad) and plated on SD agar-plates.

### Transporter identification

#### Experimental setup for *A. niger* RNA seq analysis

For pre-growth of *A. niger* NW186 and NW305, a total of 1×10^6^ spores·mL^−1^ were inoculated in 1 L Erlenmeyer shake flasks containing 200 mL medium with the following composition: 1.2 g·L^−1^ NaNO_3_, 0.5 g·L^−1^ KH_2_PO_4_, 0.2 g·L^−1^ MgSO_4_**·**7H_2_O, 40 µL·L^−1^ Vishniac solution, and supplemented with 50 g·L^−1^ (∼250 mM) glucose as carbon source, and either 1.1 mM (0.2 g·L^−1^) arginine or 5 mM (0.88 g·L^−1^) citrulline. After 24 h of pre-growth, 11 g of *A. niger* mycelium was transferred to controlled fermentors, containing the same medium as for the pre-growth. The supernatants were collected for determination of extracellular metabolite concentrations, especially carbon sources and organic acids, by high-performance liquid chromatography (HPLC) as described previously (Odoni *et al*. 2017).

#### RNA isolation, sequencing and RNA seq data processing and analysis

Total RNA extraction, RNA sequencing and RNA seq data processing and statistical analysis of the supplement experiment were performed as described in Odoni *et al*. (2017). The aligned .bam files were submitted to the European Nucleotide Archive (ENA) under the accession number PRJEB24704.

#### Shortlisting putative citrate transporter candidates

To assemble a list of likely citrate transporter candidates, the RNA seq analysis was complemented with a HMM approach and a homology based approach. HMMs were built as described (Sloothaak *et al*. 2015) from two sets of proteins (‘citrate transport’ and ‘GO:00 15137’) downloaded from the UniProt database (Bateman *et al*. 2015). The genomes used for the homology approach were downloaded from the JGI database (Nordberg *et al*. 2014): *Aspergillus kawachii* (Futagami *et al*. 2011), *Aspergillus nidulans* (Galagan *et al*. 2005; Arnaud *et al*. 2012), *Aspergillus flavus* (Arnaud *et al*. 2012), *Aspergillus fumigatus* (Nierman *et al*. 2005; Fedorova *et al*. 2008; Joardar *et al*. 2012), *Aspergillus terreus* (Arnaud *et al*. 2012), *Yarrowia lipolytica* (Dujon *et al*. 2004) and *S. cerevisiae* (Dujon *et al*. 2004). Proteins with transmembrane helix structures (from the *A. niger* ATCC 1015 *in silico* proteome) were identified using the stand-alone TMHMM 2.0 software package (Sonnhammer, Von Heijne and Krogh 1998; Krogh *et al*. 2001). Protein localisation was predicted using the stand-alone protComp software (www.softberry.com). The resulting shortlists for each approach can be found in Supplementary Data 4 (Supporting Information).

### Transporter validation

The coding sequences of two putative citrate transporter genes, with ATCC 1015 protein IDs 1165828 (*A. niger* CBS 513.88: An17g01710, *A. niger* NRRL3: 06350) and 212337 (*A. niger* CBS 513.88: An09g06720m.01, *A niger* NRRL3: 00550) were amplified from *A. niger* N402 genomic DNA. The amplified fragments were introduced into a derivative of the pYES-plasmid, whereby the GAL1 inducible promoter was replaced by the CUP1 inducible promoter (Mascorro-Gallardo, Covarrubias and Gaxiola 1996). The plasmid was PCR-amplified with the Q5 polymerase following the manufacturer's protocol.

The coding sequence of the citrate exporter candidate 1165828 (An17g01710) was cloned using Golden Gate. The Golden Gate reaction mixture was prepared as follows: 400 U of T4 DNA ligase (NEB), 10 U of BsmBI (NEB), 1.5 μL of BSA (1 mg·mL^−1^), 1.5 μL of T4 DNA ligase buffer (NEB), ∼40 fmol of each PCR product and water to bring the volume up to 15 μL. The reaction mixture was incubated in a thermocycler according to the following program: 37°C for 10 min prior to 25 cycles of digestion-ligation (37°C for 3 min, 16°C for 4 min) followed by a final digestion step (55°C for 10 min) and a heat inactivation step (80°C for 10 min). *Escherichia coli* DH5α competent cells were transformed with 1 μL of the digestion-ligation reaction, and transformants were selected on ampicillin plates. Plasmid extraction was performed using the GeneJET Plasmid Miniprep kit (Thermo Fisher Scientific, Waltham, Masschusetts/US) following the manufacturer's instructions. CENPK2–1D competent cells were transformed with a sequence-verified plasmid.

The coding sequence of the citrate exporter candidate 212337 (An09g06720m.01) was cloned using the yeast homologous recombination assembly strategy. For the in-yeast assembly, CENPK2–1D cells were directly transformed with 100 ng of the PCR-amplified pYES-CUP1 plasmid and an equimolar amount of insert, and plated on selective medium. Positive transformants were picked and grown overnight in liquid medium. Plasmids were extracted using the GeneJET Plasmid Miniprep kit following the manufacturer's instructions with minor modifications. Lysis was performed by transferring the cells to Lysing MatrixC 2 mL tubes (MP Biomedicals, Santa Ana, California/US) and homogenising them for 40 s with a FastPrep-24 from MP Biomedicals. *Escherichia coli* DH5α competent cells were transformed with the extracted plasmids for propagation, and the plasmids were then again extracted using the GeneJET Plasmid Miniprep kit following the manufacturer's instructions, and sequenced for insert verification.

For the growth experiments, yeast transformant strains were pre-grown in 15 mL SD medium supplemented with 0.0076% uracil to allow growth of the parent strain. A total of 300 μL of the pre-growth culture were inoculated in 100 mL shake flasks containing 20 mL SD medium described above, containing either 20 g·L^−1^ glucose or 20.44 g·L^−1^ glycerol. For CUP1 promoter-induction, CuSO_4_ (final concentration 1 mM) was added after 4 h of growth.

## RESULTS

### *Aspergillus niger* biomass and citrate yields under different culture conditions

We used the two *A. niger* N402 derivative strains NW305 and NW186. N402, as well as wildtype *A. niger*, has the means to produce major quantities of gluconate, oxalate and citrate, depending on the external condition, but requires pH <3 to avoid excess oxalate production in favour of increased citrate production (Ruijter, Van De Vondervoort and Visser 1999). However, both NW186 and NW305 have a mutation in glucose oxidase (*goxC17*), impairing the natural *A. niger* gluconate production. In addition, NW186 has a mutation in oxaloacetate acetylhydrolase (*prtF28*), impairing oxalate production in this strain (Ruijter, Van De Vondervoort and Visser 1999). Thus, while NW305 can produce both oxalate and citrate, but not gluconate, NW186 can produce neither gluconate nor oxalate, but retains its capacity to produce citrate, and does so even at higher pH levels (Ruijter, Van De Vondervoort and Visser 1999). In addition, NW305 and NW186 are ornithine transcarbamylase (*argB*) knock-out mutants, making them arginine auxotrophic (Lenouvel, van de Vondervoort and Visser 2002). Supplementation of the medium with either arginine or citrulline can restore growth of the *ΔargB* mutants, but we found that addition of excess (5 mM) citrulline increases total citrate production when compared to addition of (1.1 mM; ‘standard’ condition) arginine. We compared gene expression data from the experiment described above, hereafter referred to as supplement experiment, to data from one of our previous experimental setups, described in Odoni *et al*. (2017), in which increased citrate secretion was induced by iron limitation (hereafter referred to as iron experiment).

**Figure 1.**
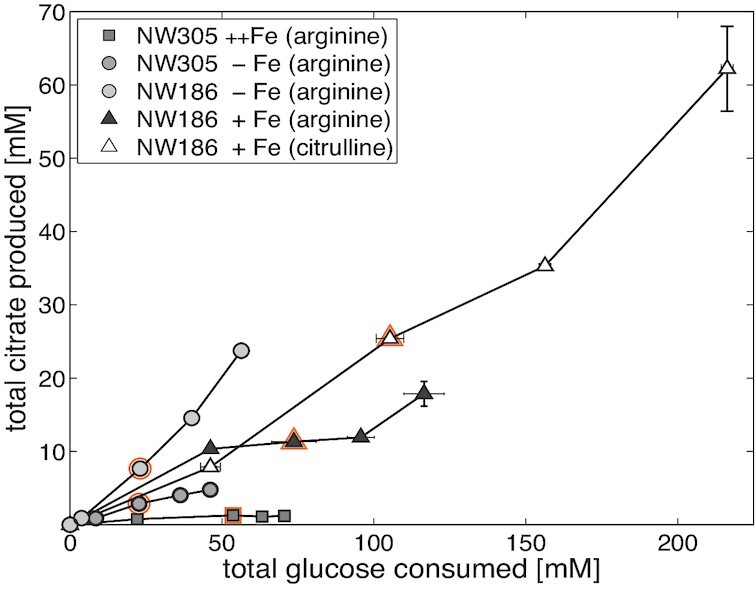
Differences in total citrate yields and production rates on glucose as carbon source. *Aspergillus niger* strains NW186 and NW305 were either grown without addition of iron (circles), or varying amounts of Fe(II)SO_4_ (triangles = 1 g·L^−1^, squares = 10 g·L^−1^) and supplemented with either arginine (filled symbols) or excess citrulline (empty symbols). Sample points for RNA extraction (t = 48 h) are marked in orange. Measurement points were taken once every 24 h and show the average of two biological replicates. Note that glucose consumption, rather than time, is plotted on the x-axis.

Citrate production yields of the considered conditions are shown in Table 1. In the supplement experiment, total citrate yield is almost doubled in NW186 + Fe_c compared to NW186 + Fe_a. In fact, and not surprisingly, excess citrulline compared to the standard amount of arginine has an overall stimulating effect on metabolism; it leads to increased glucose consumption (Fig. 1), doubled final biomass (Table 1), and increased CO_2_ production (337.79 ± 15.86 mM in NW186 + Fe_c and 265.51 ± 9.28 mM in NW186 + Fe_a). On the other hand, not adding iron to the culture medium increases the citrate per glucose production rate, but reduced glucose consumption is observed (Fig. 1), and total biomass production remains limited (Table 1).

**Table 1.** Final biomass and citrate yields of *A. niger* NW305 and NW186 grown with varying iron concentrations and/or different supplements in the medium.

Experiment	Strain	Fe	Suppl^1^	Final biomass (g)	Citrate yield	
					(g·g^−1^ biomass)	(g·g^−1^ substrate)
Iron	NW305	++	a	0.35 ± 0.02	0.67 ± 0.07	0.02 ± 0.002
Iron	NW305	−	a	0.19 ± 0.02	4.92 ± 0.35	0.11 ± 0.002
Iron	NW186	−	a	0.22 ± 1e^−03^	21.88 ± 1.51	0.46 ± 0.02
Supplement	NW186	+	a	1.87 ± 0.04	1.80 ± 0.14	0.17 ± 0.03
Supplement	NW186	+	c	3.96 ± 0.09	2.98 ± 0.35	0.31 ± 0.03

1 Suppl: supplement, with a = arginine, c = citrulline

### General transcriptome analysis

Transcriptomic data analysis of the iron experiment has already been described in Odoni *et al*. (2017). Here, we performed transcriptome analysis on the supplement experiment, i.e. NW186 supplemented with either 1.1 mM arginine or 5 mM citrulline. RNA for RNA sequencing (RNA seq) was extracted after 48 h of growth. The annotated genome of *A. niger* ATCC 1015 (Andersen *et al*. 2011) was used as reference to map the RNA seq reads (Table 2). Genes with count per million (CPM) ≥1 were considered expressed (Supplementary Data 1, Supporting Information). The supplement change from arginine to excess citrulline induces major transcriptional adaptations, and over 20% of the annotated genes are differentially expressed (Table 2; Supplementary Data 2, Supporting Information).

**Table 2.** RNA seq mapping and differential expression analysis.

	NW186 + Fe arginine		NW186 + Fe citrulline
# Reads after QC filtering	42 029 453 (1)		30 895 435 (1)
	49 141 964 (2)		68 512 663 (2)
Uniquely mapped reads	85.16% (1)		57.87% (1)
(against ATCC 1015 CDS)	68.80% (2)		68.27% (2)
# Genes expressed (CPM ≥ 1)	9620		9705
# Genes differentially expressed, log2FC threshold ≥ 0.58 (FDR ≤ 0.05)		2385	
# EC covered (mapped to KEGG pathways)		466	
# EC differentially expressed, log2FC threshold ≥ 0.58 (FDR ≤ 0.05)		173	

**Figure 2.**
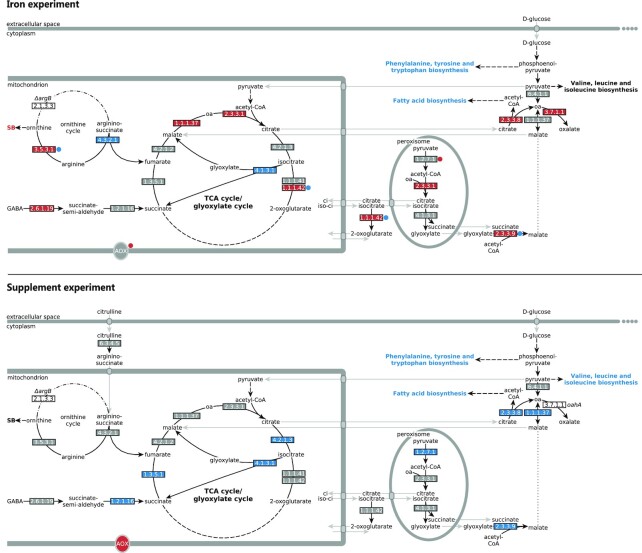
Differential expression of genes encoding enzymes involved in *A. niger* citrate metabolism. Enzymes and pathways are colour coded according to their transcriptional changes in the iron and supplement experiments. Red colour indicates higher expression in the condition with higher citrate production compared to the condition that had a lower citrate per glucose production (++Fe in the iron experiment), or total citrate production (arginine in the supplement experiment). Similarly, blue indicates lower expression in the conditions with either a higher citrate per glucose yield, or higher total citrate production. Grey indicates no significant (FDR ≤ 0.05 and log2FC ≥ 0.58) changes in expression. Abbreviations: SB = siderophore biosynthesis, oa = oxaloacetate, AOX = alternative oxidase (Aox1). Figure adapted from Odoni *et al*. (2017).

From the 466 enzyme commission (EC) numbers that have been included in KEGG maps of metabolism (Kanehisa and Goto 2000; Kanehisa *et al*. 2015), and can be found in the annotated genome of *A. niger* ATCC 1015, 37% show differential expression. Pathway enrichment analysis shows prevalence of differentially expressed genes in pathways associated to biomass formation, such as starch and sucrose metabolism, and pathways related to amino acid biosynthesis, such as phenylalanine, tyrosine and tryptophan biosynthesis and metabolism, and valine, leucine and isoleucine biosynthesis (Supplementary Data 3, Supporting Information). There is also an enrichment in differentially expressed genes related to fatty acid biosynthesis, and synthesis and degradation of ketone bodies pathways, upon addition of excess citrulline instead of arginine (Supplementary Data 3, Supporting Information). Note that, although we observed higher glucose consumption in NW186 + Fe_c, glycolysis as a pathway did not show enrichment of differentially expressed genes. Interestingly, the enzymes converting citrulline to arginine also showed no differential expression in the supplement experiment (Fig. 2; Supplementary Data 3, Supporting Information). Most of the pathways enriched in the arginine/citrulline supplement experiment (Supplementary Data 3, Supporting Information) were also enriched in the iron experiment (Odoni *et al*. 2017). Phenylalanine, tyrosine and tryptophan biosynthesis, and fatty acid biosynthesis pathways, showed similar behaviours in both experiments, and most of the enzymes in these pathways are down-regulated in the condition with higher citrate production.

Analysis of the expression of enzymes involved in citrate metabolism revealed quite a contrasting transcriptomic landscape in the two experimental setups (Fig. 2). While citrate biosynthesis genes are up-regulated in the condition corresponding to increased extracellular citrate secretion in the iron experiment, there is down-regulation of citrate metabolising enzymes in the condition corresponding to increased extracellular citrate accumulation in the arginine/citrullline supplement experiment. We also found that alternative oxidase (AOX, or non-electrogenic ubiquinol oxidase, EC 1.10.3.11) was up-regulated in the condition corresponding to the highest citrate secretion in both experimental setups (Fig. 2; Supplementary Data 2, Supporting Information).

### *Aspergillus niger* citrate exporter identification

To identify the elusive citrate exporter, we aimed to combine the gene expression data obtained from the two experimental setups with complementary bioinformatic approaches.

For one, we constructed two HMMs from multiple sequence alignments of biochemically characterised citrate transporters obtained from the UniProt database (Bateman *et al*. 2015). The sequences selected to build the models matched the terms ‘citrate transport’ and ‘GO:0015137: citrate transmembrane transporter activity’, and differences in the selected sets likely reflect differences in protein annotation in UniProt. The HMMs were used to identify and score new citrate transporter candidates in the ATCC 1015 *in silico* proteome. In addition, we identified all (predicted) plasma-membrane proteins from the ATCC 1015 *in silico* proteome and performed protein BLAST against genomes of other sequenced *Aspergilli* or yeast that are known to either produce (*A. kawachii, Y. lipolytica*) or not produce (*A. flavus*, *A. terreus, S. cerevisiae*) citrate. Finally, we also included 10 genes up- and downstream of the *A. niger* citrate synthase gene (*citA*) as putative citrate transporter candidates. We thereby followed the notion that it is not uncommon that fungal transporters are encoded by genes in close genomic proximity to the genes encoding biosynthesis of their transport target (van der Straat and de Graaff 2014).

The lists of putative citrate exporter candidates obtained from each of these steps were then filtered and ranked based on the following selection criteria (from basic to more specific importance, with putative candidate transporters not fulfilling these criteria dropping out at each step):

Average nucleotide coverage (avNtCov) ≥ 1 in all conditions.
avNtCov ≥ 50 in NW186 -Fe_a (highest citrate production rate) and NW186 +Fe_c (highest overall citrate yield).
Gene expression up-regulated in NW186 -Fe_a compared to NW305 -Fe_a.
Gene expression up-regulated in NW186 + Fe_c compared to NW186 +Fe_a.

These four points were considered the minimum requirement, and proteins had to adhere to these criteria to be considered for the HMM and proximity approaches (Supplementary Data 4, Supporting Information; sheets ‘HMM_approach’ and ‘proximity_to_citA’ approach). For a more stringent selection, applied to the proteins found based on homology in other filamentous fungi and yeasts, the following additional criteria had to be met:

Encoded protein product contains ≥1 predicted transmembrane helix domain (tmhmm).
*Aspergillus niger* protein is more similar (higher NCBI Blast ‘% identity’) to *A. kawachii* homologue than *A. flavus* and *A. terreus* homologues.
*Aspergillus niger* protein is more similar to *Y. lipolytica* homologue than to *S. cerevisiae* homologue.
Gene expression up-regulated in NW305 -Fe_a compared to NW305 ++Fe_a. (Note that this was not considered a minimal requirement, as NW305 will shift to oxalate production under iron limited conditions, and thus the direct inference on citrate production is not as clear cut as between NW186 and NW305, see also Odoni *et al*. (2017)).

The final shortlists of each approach can be found in Supplementary Data 4 (Supporting Informattion; sheets ‘HMM_approach’, ‘homology_approach’ and ‘proximity_to_*citA*’).The genes that passed the selection criteria were then ordered from high to low based on their log2 fold-changes (log2FC values) in NW186 -Fe_a vs NW305 -Fe_a (Supplementary Data 4, Supporting Information, sheet ‘all_sorted’; Table 3), as we consider this the biologically most relevant condition for *A. niger* citrate export (Odoni *et al*. 2017). As a result of our filtering and prioritisation criteria, and considering the predicted protein localisation, the protein with the *A. niger* ATCC 1015 protein ID 1165828 (*A. niger* CBS 513.88: An17g01710, *A. niger* NRRL3: 06350) is the top most likely citrate exporter candidate (Table 3). In addition, we choose another protein, with protein ID 212337 (*A. niger* CBS 513.88: An09g06720m.01, *A niger* NRRL3: 00550), as a second putative citrate exporter candidate. This protein was chosen based on being the only predicted plasma-membrane transporter candidate in the close vicinity of the *A. niger* citrate synthase gene (*citA*), although the lower expression levels suggest lesser importance as actual citrate exporter .

**Table 3.** Shortlist of average nucleotide coverage and log2 fold-changes of putative *A. niger* citrate exporter candidates found by the different approaches mentioned (Supplementary Data 4, Supporting Information; sheet ‘all_sorted’).

proteinId	tmhmm	NW305 -Fe_a	NW186 -Fe_a	log2FC	FDR	NW186 +Fe_a	NW186 +Fe_c	log2FC	FDR	NW305 -Fe_a	NW305 ++Fe_a	log2FC	FDR
1165828	12	14.61 ± 0.99	139.89 ± 22.45	3.42	0.00	34.03 ± 1.52	350.11 ± 111.78	3.30	0.00	14.61 ± 0.99	4.73 ± 0.46	−1.75	0.00
1186388	14	22.65 ± 1.00	64.92 ± 2.47	1.67	0.00	70.22 ± 5.08	250.45 ± 19.01	1.78	0.00	22.65 ± 1.00	6.90 ± 0.82	−1.88	0.00
212337	4	52.79 ± 7.20	123.91 ± 0.68	1.44	0.00	34.70 ± 1.91	116.80 ± 16.22	1.66	0.00	52.79 ± 7.20	41.65 ± 2.17	−0.44	0.48
1187835	12	24.73 ± 0.33	53.79 ± 6.51	1.26	0.00	87.03 ± 8.32	109.85 ± 46.96	0.27	0.89	24.73 ± 0.33	9.74 ± 1.07	−1.51	0.00
1155853	5	62.05 ± 1.00	132.17 ± 18.17	1.23	0.00	61.63 ± 2.29	108.63 ± 3.11	0.77	0.05	62.05 ± 1.00	61.59 ± 6.45	−0.16	0.85
1105147	14	202.52 ± 38.35	407.77 ± 8.54	1.15	0.01	34.77 ± 2.66	377.25 ± 25.34	3.42	0.00	202.52 ± 38.35	2.88 ± 0.05	−6.32	0.00
1120210	7	76.98 ± 1.19	110.49 ± 2.48	0.66	0.28	55.93 ± 0.80	65.12 ± 9.50	0.16	0.93	76.98 ± 1.19	73.30 ± 2.75	−0.24	0.75
1178251	5	1.77 ± 0.11	2.25 ± 0.34	0.62	0.55	1.34 ± 0.12	1.07 ± 0.15	−0.37	0.76	1.77 ± 0.11	2.69 ± 0.11	0.51	0.44
1137850	0	88.34 ± 5.03	118.68 ± 1.26	0.58	0.43	51.68 ± 4.67	219.35 ± 35.84	2.03	0.00	88.34 ± 5.03	26.41 ± 1.27	−1.91	0.00
1126790	1	37.23 ± 1.28	47.80 ± 1.56	0.53	0.52	54.66 ± 1.84	26.97 ± 3.30	−1.07	0.00	37.23 ± 1.28	55.51 ± 1.16	0.44	0.46
1141368	0	283.79 ± 3.09	367.47 ± 40.06	0.52	0.55	350.33 ± 11.21	420.75 ± 115.76	0.20	0.91	283.79 ± 3.09	254.87 ± 14.52	−0.33	0.63
1164596	4	196.19 ± 1.18	249.19 ± 20.70	0.50	0.59	75.44 ± 5.94	113.96 ± 9.73	0.53	0.41	196.19 ± 1.18	148.70 ± 9.82	−0.56	0.28
1146389	1	108.54 ± 4.88	138.31 ± 19.36	0.50	0.59	116.63 ± 2.51	186.06 ± 4.83	0.63	0.21	108.54 ± 4.88	108.48 ± 6.72	−0.15	0.86
1082759	1	87.33 ± 5.64	99.05 ± 0.51	0.34	0.81	64.14 ± 0.23	68.81 ± 11.91	0.05	0.98	87.33 ± 5.64	58.61 ± 6.89	−0.72	0.11
1125366	4	132.02 ± 17.77	146.93 ± 8.62	0.31	0.84	37.41 ± 1.48	109.57 ± 0.12	1.50	0.00	132.02 ± 17.77	75.85 ± 6.71	−0.92	0.02
1019516	2	144.19 ± 12.89	161.84 ± 26.21	0.31	0.85	192.89 ± 0.71	218.35 ± 9.92	0.12	0.95	144.19 ± 12.89	135.13 ± 13.09	−0.23	0.76
1104917	0	188.49 ± 34.04	207.27 ± 13.58	0.30	0.86	273.96 ± 4.26	290.30 ± 37.78	0.02	0.99	188.49 ± 34.04	106.56 ± 2.73	−0.97	0.02
1121445	11	57.61 ± 1.74	63.01 ± 2.36	0.28	0.87	72.90 ± 0.47	104.82 ± 12.52	0.46	0.54	57.61 ± 1.74	37.15 ± 4.55	−0.80	0.05
40744	3	34.08 ± 1.51	37.29 ± 3.56	0.27	0.88	132.37 ± 11.64	40.86 ± 6.76	−1.76	0.00	34.08 ± 1.51	82.38 ± 0.63	1.11	0.00
1178940	0	194.27 ± 13.70	213.06 ± 21.16	0.27	0.88	664.99 ± 5.60	612.42 ± 39.79	−0.16	0.93	194.27 ± 13.70	393.00 ± 37.13	0.85	0.05
1113466	2	338.24 ± 10.36	365.49 ± 48.70	0.24	0.91	455.53 ± 13.84	401.65 ± 53.28	−0.24	0.88	338.24 ± 10.36	423.61 ± 7.25	0.12	0.89
1156700	5	98.40 ± 3.12	101.76 ± 3.19	0.20	0.93	434.89 ± 16.18	257.81 ± 44.29	−0.80	0.07	98.40 ± 3.12	225.43 ± 20.94	1.03	0.00
1165578	12	110.33 ± 11.86	112.62 ± 0.55	0.19	0.94	107.22 ± 2.38	158.33 ± 12.11	0.51	0.44	110.33 ± 11.86	53.59 ± 0.79	−1.20	0.00
1120082	5	146.10 ± 6.70	142.51 ± 2.09	0.13	0.96	149.21 ± 1.74	172.49 ± 17.22	0.16	0.93	146.10 ± 6.70	142.19 ± 0.58	−0.18	0.82
1121621	12	509.21 ± 69.19	497.06 ± 41.68	0.13	0.96	287.69 ± 2.48	366.00 ± 34.79	0.29	0.82	509.21 ± 69.19	503.96 ± 74.66	−0.18	0.83
1147788	5	82.76 ± 8.14	76.08 ± 3.74	0.06	0.99	93.44 ± 3.39	136.89 ± 28.41	0.51	0.50	82.76 ± 8.14	72.84 ± 1.82	−0.34	0.62
1092996	4	108.82 ± 13.42	100.63 ± 3.30	0.04	0.99	103.47 ± 3.68	121.62 ± 8.56	0.18	0.92	108.82 ± 13.42	92.85 ± 0.01	−0.39	0.54
1163349	0	29.36 ± 1.78	26.80 ± 1.03	0.02	0.99	35.36 ± 1.44	32.46 ± 1.63	−0.17	0.93	29.36 ± 1.78	27.31 ± 0.94	−0.27	0.71
1141295	2	197.00 ± 8.52	180.50 ± 1.00	0.01	1.00	112.86 ± 4.46	136.16 ± 7.77	0.20	0.91	197.00 ± 8.52	150.75 ± 3.28	−0.55	0.29
1103501	14	140.87 ± 5.80	128.08 ± 13.18	0.01	1.00	96.46 ± 6.34	206.50 ± 33.35	1.05	0.00	140.87 ± 5.80	3.30 ± 0.06	−5.56	0.00
1172271	1	134.47 ± 5.07	121.28 ± 1.70	0.00	1.00	66.79 ± 2.94	82.65 ± 12.41	0.24	0.87	134.47 ± 5.07	107.55 ± 1.94	−0.47	0.42
1219631	0	1.92 ± 0.37	1.56 ± 0.08	−0.01	1.00	4.76 ± 0.47	5.85 ± 1.00	0.28	0.85	1.92 ± 0.37	1.64 ± 0.37	−0.30	0.72
177364	1	2.82 ± 0.18	2.61 ± 0.00	−0.03	0.99	1.88 ± 0.07	1.87 ± 0.32	0.01	1.00	2.82 ± 0.18	1.46 ± 0.05	−1.04	0.03
1137953	2	40.68 ± 3.74	34.00 ± 3.57	−0.11	0.97	141.12 ± 2.32	63.58 ± 6.84	−1.21	0.00	40.68 ± 3.74	85.18 ± 6.98	0.92	0.02
1117050	3	40.36 ± 1.55	32.08 ± 4.00	−0.16	0.95	46.35 ± 3.98	53.35 ± 4.46	0.15	0.94	40.36 ± 1.55	21.28 ± 0.77	−1.07	0.00
1141371	0	1571.19 ± 246.87	1249.67 ± 120.07	−0.17	0.95	551.49 ± 6.68	518.11 ± 5.14	−0.15	0.94	1571.19 ± 246.87	740.57 ± 40.61	−1.22	0.00
1147568	4	7482.49 ± 509.41	5782.62 ± 105.11	−0.22	0.92	3164.92 ± 14.66	3994.04 ± 231.82	0.28	0.84	7482.49 ± 509.41	6622.43 ± 20.86	−0.36	0.60
1181871	12	49.66 ± 1.89	36.63 ± 0.94	−0.29	0.87	37.12 ± 0.83	30.34 ± 6.81	−0.36	0.74	49.66 ± 1.89	35.43 ± 0.48	−0.65	0.16
1209512	0	50.80 ± 5.25	36.89 ± 3.37	−0.30	0.85	89.37 ± 2.45	60.30 ± 0.80	−0.61	0.25	50.80 ± 5.25	87.27 ± 4.03	0.62	0.20
1011311	0	55.14 ± 2.22	38.64 ± 6.57	−0.36	0.79	119.93 ± 7.34	98.35 ± 0.78	−0.34	0.76	55.14 ± 2.22	88.47 ± 3.26	0.55	0.28
1168387	0	60.92 ± 2.05	40.64 ± 0.63	−0.45	0.66	91.23 ± 0.81	80.83 ± 10.18	−0.22	0.89	60.92 ± 2.05	59.55 ± 5.52	−0.22	0.78
1141800	1	43.05 ± 0.75	25.30 ± 1.81	−0.61	0.36	120.51 ± 4.34	73.38 ± 12.23	−0.79	0.07	43.05 ± 0.75	59.09 ± 1.45	0.35	0.60
1169809	12	113.09 ± 7.38	58.75 ± 7.85	−0.79	0.14	195.35 ± 3.82	323.04 ± 63.23	0.66	0.24	113.09 ± 7.38	140.31 ± 24.63	0.18	0.83
1164227	2	43.66 ± 2.41	19.30 ± 1.11	−1.01	0.01	124.93 ± 0.50	30.60 ± 1.03	−2.08	0.00	43.66 ± 2.41	100.11 ± 12.83	1.04	0.00
1148364	0	66.99 ± 4.34	29.59 ± 0.47	−1.02	0.01	19.77 ± 1.34	31.72 ± 6.23	0.60	0.35	66.99 ± 4.34	21.58 ± 1.63	−1.75	0.00
1222809	0	172.06 ± 39.87	70.73 ± 9.77	−1.10	0.05	52.51 ± 0.89	74.55 ± 15.51	0.44	0.61	172.06 ± 39.87	14.50 ± 1.37	−3.74	0.00
1115499	0	41.79 ± 0.54	14.21 ± 0.30	−1.40	0.00	25.87 ± 1.86	19.65 ± 1.58	−0.44	0.59	41.79 ± 0.54	30.57 ± 3.99	−0.60	0.24
1146086	1	73.65 ± 8.51	23.91 ± 0.59	−1.47	0.00	61.61 ± 1.55	63.36 ± 9.68	0.00	1.00	73.65 ± 8.51	26.66 ± 0.81	−1.62	0.00
37369	0	0.03 ± 0.02	− ± −	#N/A	#N/A	0.03 ± 0.02	0.02 ± 0.01	#N/A	#N/A	0.03 ± 0.02	− ± −	#N/A	#N/A
1178960	0	0.23 ± 0.05	0.46 ± 0.02	#N/A	#N/A	0.71 ± 0.07	0.50 ± 0.00	#N/A	#N/A	0.23 ± 0.05	0.73 ± 0.11	#N/A	#N/A

### *In vivo* validation of selected citrate exporter candidates in *S. cerevisiae*

**Figure 3.**
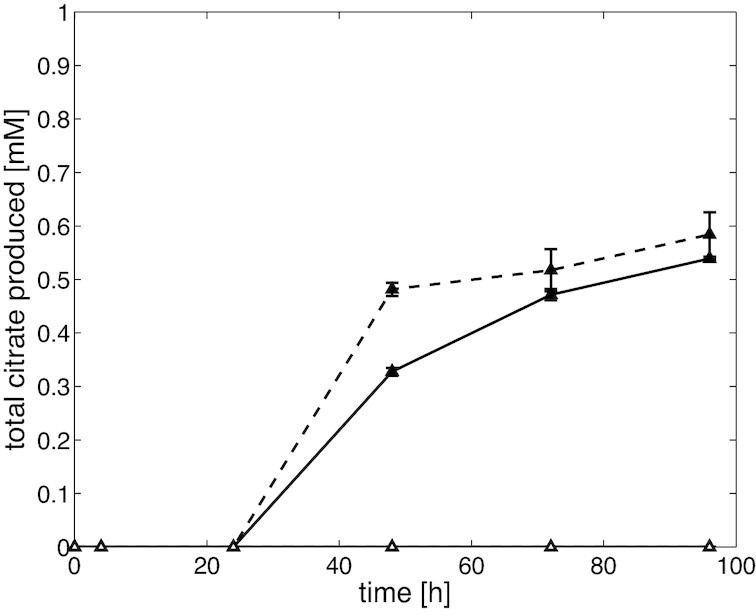
Citrate secretion in *S. cerevisiae* transformed with CexA. Solid line = grown on glucose, dashed line = grown on glycerol, filled symbols = induced and empty symbols = non-induced. Note that there was no measurable extracellular citrate accumulation in the untransformed parent strain. Measurements indicate the average of two biological replicates.

*Saccharomyces cerevisiae* was transformed with plasmids containing either one of the putative citrate exporter candidate genes under the control of a copper inducible promoter (CUP1). In the first experiment, we added CuSO_4_ after 4 h of growth to the medium of all strains to induce expression of the transporter proteins, and found that neither the untransformed control strain, nor the strain transformed with the protein 212337 (An09g06720m.01) encoding gene secreted any citrate. However, under these exact same conditions, the strain transformed with the protein 1165828 (An17g01710) encoding gene accumulated a small amount of extracellular citrate after induction of gene expression with CuSO_4_, indicating that this could be a citrate exporter. To further verify our initial results, we grew *S. cerevisiae* transformed with the promising citrate exporter gene with either glucose or glycerol in the medium, and either did or did not induce expression with CuSO_4_. Confirming our initial observations, we found accumulation of citrate in the extracellular medium in *S. cerevisiae* when heterologous gene expression was induced (Fig. 3). Again, no citrate accumulation was observed in the extracellular medium of the untransformed control strain, irrespective of whether or not we added CuSO_4_ to the medium, excluding the possibility that the observed effect was merely a result of intracellularly accumulated citrate due to different media conditions. These data reconfirm that the *A. niger* transporter protein identified as top most likely citrate exporter candidate (Table 3) is indeed a citrate exporter. When initially making our findings publicly available (Odoni *et al*. 2018) we named this newly identified *A. niger* citrate exporter ‘CitT’ (**Cit**rate **T**ransporter). However, Steiger *et al*., who independently recognised this transporter as *A. niger* citrate exporter using a homology approach, argue that our name for this exporter can lead to confusion with the *citT* gene in *E. coli*, which encodes a citrate/succinate antiporter (Steiger *et al*. 2019). Therefore, adhering to the nomenclature given by (Steiger *et al*. 2019), we refer to this transporter protein as CexA.

## DISCUSSION

Combining the expression values from our experimental setups with the complementary *in silico* homology approach ultimately led to the identification of the *A. niger* citrate exporter CexA (Table 3). Notably, the only other successful approach to identify the *A. niger* citrate exporter thus far was also *via* a homology approach, using an itaconate transporter from *Ustilago Maydis* as template (Steiger *et al*. 2019). The rationale behind using this template was that itaconate and citrate share a considerable amount of chemical similarity to justify transport *via* the same system (Steiger *et al*. 2019). This rationale proved to be successful, and we have also previously advocated the use of sequence similarity to identify new transporters of a given substrate of interest (Sloothaak *et al*. 2015, 2016). However, these approaches rely on the availability of the appropriate templates for any given substrate, which might not always be the case (Sloothaak *et al*. 2015).

Here, we used a homology approach, which we specifically conceived for the scenario in which it is not known which transporters are responsible for citrate export in the organisms used for comparison. Our only assumption was that the presence of a transporter performing this function is either more (*A. kawachii, Y. lipolytica*) or less (*A. flavus*, *A. terreus, S. cerevisiae*) likely. However, these organisms might share a variety of homologous proteins. Thus, sorting the list of putative citrate transporter proteins based on their log2FC values in the experimental setup in which we hypothesise that differences in citrate secretion are crucial for survival of the fungus was key (Table 3).

Interestingly, CexA has not been listed as putative citrate exporter candidate using a transcriptomics approach in the citrate producing strain H915–1 (Yin *et al*. 2017), although *cexA* (An17g01710) gene expression was found to be highly induced by sucrose (Yuan *et al*. 2008). This suggest different transcriptional regulation of citrate secretion under different citrate producing conditions. Indeed, the contrasting transcriptomic landscapes of enzymes involved in citrate metabolism (Fig. 2) imply very distinct molecular mechanisms underlying the phenotype of increased citrate accumulation in the two experimental setups used for this study.

In the supplement experiment, we observed a higher glucose consumption rate when NW186 was supplemented with excess citrulline (Fig. 1), although this was not reflected in up-regulation of glycolysis at transcript level (Supplementary Data 2, Supporting Information). A high glucose consumption rate results in high glycolytic flux that will lead to excess NADH, the turn-over of which might be limited by the capacity of further biosynthetic processes (Karaffa and Kubicek 2003). The observed down-regulation of TCA cycle enzymes downstream of citrate in NW186 +Fe_c compared to NW186 +Fe_a prevents citrate to be further metabolised, which might be a mechanism to prevent further generation of NADH (Gallmetzer and Burgstaller 2002). This mechanism represents citrate production *via* overflow metabolism in its most classical form (Legiša and Mattey 2007).

*Aspergillus niger* strains used in academia and industry are subject to conditions they would not usually encounter in nature, and the surplus of glucose or another carbon source encountered by the fungus would almost always suggest citrate production *via* overflow metabolism. Given these conditions, it has been reported that alternative oxidase (also non-electrogenic ubiquinol oxidase, or Aox1) is required for efficient *A. niger* citrate production to avoid excess production of ATP (Ruijter, Kubicek and Visser 2002). Aox1 in *A. niger* effectively decouples ATP generation and NADH re-oxidation, as the reduction of molecular oxygen to water by alternative oxidase bypasses proton translocation *via* the oxidative phosphorylation complexes III and IV, resulting in a lower ATP yield (Joseph-Horne, Hollomon and Wood 2001).

As expected in a condition of metabolic overflow, up-regulation of *aox1* was accompanied with down-regulation of cytochrome-dependent respiratory enzymes when supplementing NW186 with excess citrulline, indicating a switch from cytochrome-dependent respiration to alternative respiration (Karaffa and Kubicek 2003). However, in the iron experiment, the observed up-regulation of *aox1* was not accompanied with down-regulation of cytochrome-dependent respiratory enzymes (Supplementary Data 2, Supporting Information), possibly indicating that no such switch takes place. In a different study, it was found that incubating detached roots of the plant *Poa annoa* with citrate increased protein concentration of alternative oxidase without actually increasing the activity of the alternative respiration pathway itself (Millenaar 2002). The authors of the study hypothesised that the chelating properties of citrate might lead to the withdrawal of the Fe in the active centre of alternative oxidase, thereby rendering the protein inactive and evoking increased transcription to compensate for the inactive protein. In a similar line of reasoning, the transcriptional increase of *aox1* observed in the iron experiment might be a futile attempt to counteract high intracellular accumulation of citrate preceding its secretion in NW186 -Fe_a.

Due to the chelating properties of citrate and other organic acids, we have previously discussed up-regulation of *A. niger* citrate biosynthesis and secretion as ‘active’ response to overcome iron limitation (Odoni *et al*. 2017). Remarkably, CexA, which seems to be the only active citrate exporter based on the inability of *A. niger cexA* KO mutants to secrete citrate (Steiger *et al*. 2019), is a major facilitator protein belonging to the subfamily of H^+^-drug antiporters. In plants, these transporters are used for (trace) metal homeostasis (Magalhaes 2010; Remy and Duque 2014), relating these findings back to our notion that a probable biological role for citrate is to act as an *A. niger* iron siderophore. Another conceivable role for citrate is to aid in the hydrolysis of sucrose to glucose and fructose, a process citrate can be used for (Lowe 1937). Again, the need to use citrate to fulfil a biological function extracellularly offers a possible explanation why sucrose is such a good carbon source for triggering *A. niger* citrate secretion (Hossain, Brooks and Maddox 1984), and also why *cexA* (An17g01710) gene expression was highly induced on sucrose (Yuan *et al*. 2008).

## Supplementary Material

Supplemental Files
